# A study protocol for a therapist guided digital cognitive-behavioral therapy for children with avoidant/restrictive food intake disorder and their parents

**DOI:** 10.3389/fpsyt.2026.1743480

**Published:** 2026-02-11

**Authors:** Julia E. Engelkamp, Lara-Marie Koeder, Manuela Dalholf, Jennifer J. Thomas, Kamryn T. Eddy, Andrea S. Hartmann

**Affiliations:** 1 Department of Psychology, Clinical Psychology and Psychotherapy of Childhood and Adolescence, Konstanz University, Konstanz, Germany; 2 Eating Disorders Clinical and Research Program, Massachusetts General Hospital, Boston, MA, United States; 3 Department of Psychiatry, Harvard Medical School, Boston, MA, United States; 4 Division of Child and Adolescent Psychiatry, AMC Department of Psychiatry, Massachusetts General Brigham, Boston, MA, United States

**Keywords:** avoidant restrictive food intake disorder, CBT-AR, children, digital intervention, sensory sensitivity, treatment outcomes, virtual treatment

## Abstract

**Background:**

Avoidant/restrictive food intake disorder (ARFID) is an eating disorder prevalent in children and associated with several health and psychosocial impairments. Despite its prevalence, limited treatment services are available and children with ARFID often do not receive specialized care. Cognitive-behavioral therapy has shown feasibility in face-to-face trials supporting proof-of-concept. Digital interventions could increase treatment accessibility in this population.

**Methods:**

In a single-armed intervention trial (*N* = 40) children aged 8–14 years with a primary diagnosis of ARFID and their caregivers will receive a digital cognitive-behavioral therapy. The intervention consists of a 12-session program, combining video therapy sessions with children and caregivers and self-guided modules. Assessments occur at baseline, mid-treatment, post-treatment, and 4-week follow-up with additional symptom monitoring questionnaires per session. Primary outcomes include feasibility, recruitment and retention rate. Secondary outcomes include ARFID symptoms (e.g., Eating Disorder Examination ARFID module, Nine Item Avoidant/Restrictive Food Intake Disorder screen), food neophobia (Food Neophobia Scale) and quality of life (Generic Quality of Life Instrument for Children-Revised).

**Discussion:**

This study evaluates the feasibility and proof of concept of a therapist-guided digital intervention for ARFID. If feasible and acceptable, the digital intervention may help to increase treatment access for children with ARFID in German-speaking countries. Furthermore, findings can inform future larger-scale randomized control trials and the development of digital interventions for ARFID.

**Clinical Trial Registration:**

https://drks.de/search/de/trial/DRKS00038026/details, identifier DRKS00038026.

## 1 Introduction

Avoidant/restrictive food intake disorder is a feeding and eating disorder associated with several negative health consequences (e.g., nutritional deficiencies and low bone health) (1). According to the *Diagnostic and Statistical Manual of Mental Disorders Fifth Edition* (*DSM–5*) (2) the diagnostic criteria include significant weight loss or failure to achieve expected growth, nutritional deficiencies, dependence on enteral feeding or nutritional supplements, and/or marked impairment in psychosocial functioning. ARFID is most common in children and early adolescents with prevalence ranging from 0.3% to 15.5% in community and school samples and from 5% to 22.5% in pediatric eating disorder services (3). The onset is younger compared to other restrictive eating disorders such as anorexia nervosa (3). ARFID is heterogeneous with variable presentations (sensory sensitivity, lack of interest in eating or food, or fear of negative consequences of eating) (2). Studies have shown that 61-77% of children and adolescents with ARFID present with sensory sensitivity as at least one of their presentations (4–6). Despite its prevalence in childhood and associated health consequences, the majority of individuals with eating disorders, including ARFID, do not receive specialized treatment services (7, 8).

Various interventions have been developed to treat ARFID in children and adolescents. While fully powered randomized control trials are currently lacking, pilot studies suggest that outpatient therapies that involve families demonstrate feasibility and acceptability for pediatric patients (9–12). In a pilot study, a technology-supported behavioral day treatment program for young children aged 1–6 years increased the number of bites accepted by the children from pre- to post-intervention (9). For slightly older children, aged 6–14 years, Shimshoni, Silverman (10) developed the ARFID Supportive Parenting for Anxious Childhood Emotions (SPACE) to target parental accommodation to the child’s eating behavior. Pilot study results revealed high satisfaction and a reduction in ARFID symptoms following treatment (10). Furthermore, family-based treatment adapted for ARFID (FBT-ARFID) of children aged 5 to 12 years showed promising results in a feasibility randomized control trial. The findings showed that families accepted FBT-ARFID and demonstrated medium to large effect size differences for clinical severity supported FBT compared to treatment as usual (11).

Cognitive behavioral therapy interventions have also shown promise for treating ARFID. A clinical case series of children ages 10–18 years with ARFID who received CBT for ARFID delivered in a day treatment setting demonstrated that 10 out of 11 children were in remission at three-month follow-up (13). Furthermore, Thomas & Eddy (2019) developed an outpatient manualized Cognitive-Behavioral Therapy for Avoidant/restrictive food intake disorder (CBT-AR (14)) that they and others have now tested in two open trials (12, 15) and several case studies (16–18). CBT-AR was developed for children, adolescents and adults from the age of 10 years and leverages food exposure as the key intervention. It is delivered in an individual or family supported format, with the latter being recommended for children and adolescents under the age of 16 or older adolescents who are underweight (14). Two studies showed that CBT-AR is feasible as well as acceptable in both pediatric (15) and adult (12) patients, demonstrating evidence for proof of concept (12, 15). In the pediatric sample, 70% achieved remission at post treatment and patients incorporated an average of 16.7 new foods in their diet (15). Moreover, CBT-AR suggests different exposure-based treatment strategies tailored to the three different presentations of ARFID (sensory sensitivity, lack of interest in eating or food or fear of negative consequences), making it flexible to adapt to patient specific presentations. Importantly, patients with sensory sensitivity showed significantly greater improvement when receiving the targeted intervention for this presentations compared to those who did not receive the presentation-specific intervention in a CBT-AR intervention study (19). To our knowledge, none of the previous cited interventions have been evaluated in German speaking countries where ARFID prevalence has been established (4, 20, 21), and specialized treatment services are currently lacking.

Although these novel treatments for ARFID are under study and increasingly utilized in the community, most individuals with ARFID in the community struggle to access these evidence-based approaches. Indeed, individuals with ARFID face various health care barriers and report long health care journeys before reaching specialized health care services (22–25). This delay may be attributed to health care professionals and clinicians reporting difficulties in identifying ARFID and the lack of clear treatment recommendations. This highlights the need for more intervention to identify accessible evidence based treatments (24, 26). Furthermore, an interview-based life history study of families’ experiences in the healthcare system revealed that shame experienced by parents and perceived blaming by healthcare providers might hinder treatment access (23). Additionally, the lack of specialized treatment options in non-urban areas (23) and family time constraints may further decrease accessibility (27).

One potential solution to increase accessibility and flexibility of treatment are digital health intervention (i.e., use of technology to support health) (7, 28). According to the World Health Organization digital intervention describes the use of digital technologies to enhance health fields, including ehealth (i.e. the use information and communications technology in health) or other technology such as artificial intelligence (7). Digital health interventions can provide a more private treatment access than standard face to face therapy, thereby reducing shame as a treatment barrier (29). Moreover, they have been shown to be feasible and effective for children and adolescents with eating disorders (30). Many of the ARFID therapies have been successfully delivered via video therapy in the previously mentioned trials (10, 16, 27, 31). Beyond video therapy, digital ehealth interventions also include web-based delivery of information in interactive modules or apps. These self-guided or asynchronous therapist-guided modules allow for flexible and individualized timing of the intervention without relying on availability of a study therapist. In a non-clinical population, nutritional interventions delivered to parents have been shown to improve nutritional intake (e.g. fruit and vegetable consumption) in children (32). However, to our knowledge, digital interventions including web-based delivery of sessions for ARFID are currently lacking.

Therefore, the aim of the present pilot intervention study will be to evaluate the feasibility and proof-of-concept of a CBT-AR based digital intervention “ExpeditionFood” for children and their parents/caregiver(s). Since sensory sensitivity is the most prevalent presentation of ARFID (4–6), the digital intervention will focus on individuals with sensory sensitivity. Based on the reported high prevalence in children ages 8–14 years (3, 4), the study will include children in this age range. Consequently, this study will simultaneously evaluate the feasibility of CBT-AR for a slightly younger age than in previous CBT AR trials (aged >10 years). Additionally, none of the previous cited interventions have been evaluated in German speaking countries where ARFID prevalence has been established (4, 20, 21). Hence, the primary aim of this study is to assess feasibility in German speaking countries and identify effect sizes for future randomized control trial. We hypothesize that a digital family supported intervention for children with ARFID will be feasible with acceptable recruitment and dropout rates and accepted by children and caregivers. Additionally, we hypothesize that the digital intervention will reduce ARFID symptoms, food neophobia and psychosocial impairment from pre- to post-intervention and will also increase quality of life. Finally, we will investigate if these effects are maintained until the four-week follow-up.

## 2 Methods

### 2.1 Design

We will conduct a single-arm clinical trial to evaluate the feasibility of a digital therapist-guided CBT-AR intervention for children with ARFID and their parents/guardians. The 12-week intervention will be based on the original manualized CBT-AR intervention translated to German. The CBT-AR specific food exposure strategies for sensory sensitivity will be delivered via a mixture of video therapy sessions and self-guided modules (14). Based on the reported high prevalence in children ages 8–14 years (3, 4), the study will focus on this age range. As CBT-AR has been developed from the age of 10 years on, we made a few adaptions to adopt for the slightly younger age range. Firstly, to decrease treatment burden on the children with often lower treatment motivation (23), we will offer self-guided sessions for parents/guardians and the child will join for the family supported video therapy sessions only. Secondly, we developed psychoeducational materials for the different ages with psychoeducational stories to enhance engagement of the younger children and different aged case examples. We will assess primary and secondary outcomes at pre-assessment/baseline, mid-assessment, post-assessment and 4-week follow up. Parents/guardians and children will be gifted a small coupon for completing the assessments. No incentive will be provided for participation in the intervention itself. Furthermore, the study is registered in the German Registry of Clinical Studies (Deutsches Register Klinischer Studien: DRKS00038026).

### 2.2 Inclusion and exclusion criteria

Inclusion criteria include an age between 8 to 14 years with a primary diagnosis of ARFID and a primary presentation of sensory sensitivity as indicated by the ARFID module for the Eating Disorder Examination (ChEDE) (33). Children may have additional other phenotypes of ARFID or comorbidities. The ChEDE assesses all three presentations of ARFID and is currently the only available validated expert-interview in German. Exclusion criteria for the children include severe underweight (<3rd percentile); reliance on tube feeding; medical conditions prohibiting current treatment indicated by parents/guardians or pediatric doctor; changes in psychopharmacological treatment in the previous two months; comorbidities that would reduce the appropriateness of the intervention (e.g., psychotic disorder); acute suicidality; substance abuse; or concurrent psychotherapy, dietetic/nutritionist consultation or occupational therapy. In the case of concurrent treatment, patients can participate if they and their present health care provider prefer study participation and agree to pause any concurrent treatment. Lastly both the child and the caregiver have to be sufficient in German to understand the intervention content. Other than that, no additional exclusion criteria for caregivers apply.

### 2.3 Recruitment and sample size

Recruitment will take place in Germany, Switzerland, Austria where German is (at least one of) the officially spoken languages. We will recruit families via the study’s website, social media (e.g., Instagram, Facebook), flyers, articles in our university press, and advertisements provided to outpatient health care providers affiliated with Konstanz University.

We conducted a power analysis with G*Power (version 3.1) to estimate a sample size, and also considered the sample sizes used in previous one-arm CBT-AR trials (10, 11, 15, 34). Hence, we based our power analysis on a = 0.05 and 1-b = 0.95, and a medium-sized effect size of f = 0.6, which resulted in a sample size of n = 32. To account for an expected dropout rate of 20-30% (15), we will aim to recruit *N* = 40. However, as the primary aim is to assess overall feasibility and acceptability and our main secondary aim is to identify effect sizes for randomized control trials, we are aware that depending on dropout rates the power might not be sufficient to make conclusions about the efficacy.

### 2.4 Assessment of eligibility

Interested parents/guardians will be informed about the study procedure and complete a standardized pre-screening form. The questionnaire will be delivered in German over the software Qualtrics and assesses potential eligibility (e.g., age criteria and symptoms of ARFID). Since ARFID questionnaires are currently not validated in German and standardized screening cut offs are lacking, we translated the Nine Item Avoidant/Restrictive Food Intake disorder screen (NIAS) with the authors permission using forward-backward translation. We will define the presence of sensory sensitivity based on the NIAS scores > 9 on sensory sensitivity (35), as suggested by the English validated version, as standardized screening cut offs in Germany are lacking. If the screening suggests the presence of the inclusion criteria, we will schedule a video-based diagnostic interview. Parents and their child will be eligible if a primary diagnosis of ARFID with sensory sensitivity is confirmed according to the DSM-5-TR criteria based on the ARFID module for the Eating Disorder Examination (33). Additionally, we will conduct the Diagnostic Interview for Mental Disorders for Children and Adolescents (Kinder-DIPS) (36, 37) to assess any comorbid mental disorder and ask for any diagnosed disorders (e.g., autism). After the interview, we will ask participants to obtain a medical statement from their pediatrician to ensure that no medical reasons prohibit study participation. In case of exclusion at any stage, we support excluded participants to find an alternative treatment (e.g., by providing information about treatment options and health care service in Germany, resources for ARFID) to acknowledge and decrease family burden.

### 2.5 Ethical approval and informed consent

The ethical committee of Konstanz University provided ethical approval for the study (IRB 25-2024). The diagnostician will retrieve written informed consent from all parents/legal guardians prior to the interviews.

### 2.6 Intervention

We have made few adaptations from the manualized CBT-AR (14) to ensure suitability for digital delivery to parents and translated the intervention to German. The ExpeditionFood intervention consists of 12 sessions including eight self-guided sessions and four video therapy sessions with a study therapist. The self-guided sessions will be delivered to the parents/guardians and take about 30-60min each. The video therapy sessions will be conducted with both parents/guardians and the child with a duration of approximately 50min-60min. Additionally, psychoeducation materials for children will be provided for each session (e.g., brief summaries, case examples and psychoeducational stories). We will recommend that families complete one session per week resulting in a 12-week program. Parents/guardians will access the psychoeducational materials, video call sessions and questionnaires via the ehealth platform Curafida (ZTM Bad Kissingen GmbH, 2025). The platform can be accessed via a web browser or the ExpeditionFood app. Psychoeducational materials were created in the software Articulate Rise 360 (Articulate Global LLC., 2024). Additionally, patients can download additional materials, track their progress and write to the study therapist over chat. The study therapists will include a clinical psychologist (third author) and clinical psychologist in training (first author) under the supervision by the last author (a clinical psychologist with >15 years’ experience assessing and treating eating disorders).

Similar to the CBT-AR, the ExpeditionFood intervention consists of 4 stages, but it focuses only on sensory sensitivity as a proposed mechanism and not on weight gain (underweight participants are excluded). Therefore, additional weight gain sessions or sessions for other maintaining mechanisms are not needed. Secondly, to prioritize food exposure and behavioural change early in the intervention, we shortened CBT-AR stage 1 and 2 to one session each. In line with the CBT-AR, stage 1 will provide psychoeducation on ARFID, and stage 2 will provide psychoeducation on nutrition. In stage 3, families will conduct food exposures with their child—first in a learning phase (3a) and later in a repeated in a practice phase (3b). Stage 4 will address relapse prevention. **Table 1** depicts a detailed overview of the ExpeditionFood intervention.

**Table 1:** Structure and content of the intervention.

Stage	Session	Format	Content	Homework
1	1	Clinican-delivered video-therapy	Psychoeducation: Symptoms of ARFID, CBT-AR treatment structure, development of individual ARFID formulation	Starting a parent-monitored food diary, adopting a regular eating schedule, establishing first small changes in eating behaviors
2	2	Self-guided session	Psychoeducation on balanced diet, risk of nutritional deficiencies, planning food exposure tasting session	Identifying underrepresentation of food groups in child’s diet, choosing foods for food exposure. Optional: Creating a reward schedule
3a	3	Clinican-delivered video-therapy	Food-Exposure tasting session	Food-Exposure tasting session and starting an exposure tracker
3a	4	Self-guided session	Additional tips for food-exposure sessions; strategies of integrating new foods (part 1)	Food-Exposure tasting session, tracking of exposure and integrations of foods
3a	5	Clinican-delivered video-therapy	Food-Exposure tasting session	Food-Exposure tasting session, tracking of exposure and integrations of foods
3a	6	Self-guided session	Food-Exposure tasting session, strategies of integrating new foods	Food-Exposure tasting session, tracking of exposure and integrations of foods
3a	7	Clinican-delivered video-therapy	Food exposure tasting session and troubleshooting of integrating new foods	Food-Exposure tasting session, tracking of exposure and integrations of foods
3b	8-11	Self-guided session and chat feedback	Food exposure tasting sessions and integration of accepted new foods	Food-Exposure tasting session, tracking of exposure and integrations of foods
4	12	Clinican-delivered video-therapy	Planning of further recovery steps; potential difficulties and relapse prevention	

ARFID, avoidant/restrictive food intake disorder. CBT-AR, Cognitive-Behavioral Therapy for Avoidant/restrictive food intake disorder.

At the beginning of each self-guided session, parents will fill out a brief questionnaire prompting them to reflect about the previous week They will have the chance to report difficulties, brainstorm potential solutions, and formulate questions for the study therapist. We will present psychoeducational materials in written formats including interactive functions (e.g., drop down menus) and engaging pictures. Psychoeducational materials are divided into subsections with a progress tracker (i.e., percentage of completion). Sections include “important” sections highlighting key aspects, “good to know” sections with additional information, and sections with relevant case examples. The intervention will encourage parents to apply the learned knowledge directly in daily life by setting concrete goals at the end of each session. Additionally, we will provide downloadable materials including a handout summary of the presented materials and homework sheets. For the homework and tracking (e.g., food diary, tracking of exposure section), parents can choose to either directly track food exposures in the platform or by filling out printable worksheets and uploading them at the end of each week.

In stage 3b, the practice phase, the study therapist will provide written feedback via chat on food exposure progress once weekly. Brief self-guided sessions will remind parents weekly of important strategies and tips without any new psychoeducational content. We will explain to parents that the chat will only be monitored in stage 3b and can otherwise only be used to upload homework assignments.

The video sessions will follow a similar structure, starting with an agenda, homework review, and troubleshooting. Afterwards, the therapist will present new psychoeducational materials or guide the family to conduct an in-session food exposure.

To support caregivers during the intervention and to decrease family burden (23, 38), we adopt the CBT-AR stance (i.e., non-blaming and empathic (12)) in all interactions, provide options for caregivers to add agenda points, such as concerns or questions, in video therapy sessions and allow for time flexibility in self-guided modules to decrease any extra stress associated with treatment.

### 2.7 Assessment and data management

We will assess at screening (t0), baseline (t1), mid-intervention after completion of session 6(t2), post-intervention after completion of session 12(t3) and 4-week follow-up (t4). We will use German translations of all questionnaires. Additionally, parents/guardians will complete brief weekly measures after every session. Self-report data will be assessed via Qualtrics (i.e., for outcome measures, Qualtrics, 2025) and participants will be asked to provide only their assigned participant code when answering the questions. Only the internal study team will have access to the key file in digital password protected folder to link the participants code to the names. Additionally, the secure health platform Curafida (ZTM Bad Kissingen GmbH, 2025) will integrate the software MyMedax for content and reflection questions in self-guided sessions. We will use the secure video call software webPRAX for video call sessions (Healthy Projects GmbH, n.d.). We created a data protection policy and a register of all processing activities with the legal office of Konstanz University for this study. Moreover, a data protection and confidentiality agreement will be in place between the company of the health platform ZTM Bad Kissingen GmbH and the University of Konstanz.

### 2.8 Primary outcome measures

#### 2.8.1 Feasibility and acceptability

We will assess feasibility via a retention rate. Rates ≥ 70% from pre- to post-assessment will be considered successful (39, 40). Another indicator will be the number of adverse events reported by the parents or child. Parents/guardians will be asked to report potential adverse events at the end of each session based on the recommendation for internet-intervention (41). Based on previous feasibility studies in children with ARFID we will consider a recruitment rate of ≥1 patient per month as feasible (11, 15). Additionally, based on similar research studies we will apply the following benchmarks enrolment (≥75% eligible participants enrol in the intervention). As an indicator of participants engagement, we will assess the number of times patients tasted new foods over the course of the intervention and how many different foods they tasted per week. Furthermore, we will report the average time needed to complete the intervention.

Additionally, we will ask parents and children at mid- and post treatment to rate the perceived feasibility of different intervention parts (e.g., weekly psychoeducational materials, video therapy sessions). These include ratings on the feasibility of time and effort needed for the intervention, understanding of the materials and satiation with technical quality of the platform on a 6-Point Likert scale (0 = completely agree to 1 = completely disagree). We will also measure parents’ acceptability of the intervention will by the Credibility/Expectancy Questionnaire (CEQ) (42) completed by parents and a single-item for the children.

### 2.9 Secondary outcomes

#### 2.9.1 Clinical outcomes

As proof of concept, we will assess ARFID symptoms. We will therefore administer the Nine Item Avoidant/Restrictive Food Intake disorder screen (NIAS (35, 43)) and a shortened version of the Food Neophobia Scale (FNS (44, 45)) at each time point. Additionally, we will use the parent- and child- version of the ARFID module of the ChEDE (33) to assess the fulfilment of ARFID criteria and to assess ARFID presentation. The subscales psychosocial impairments and sensory sensitivity will be used as an indicator for reduction in ARFID symptoms according to expert-ratings. Additionally, we will examine exploratory the subscales ChEDE lack of interest and fear of negative consequences to examine if changes in sensory sensitivity also affect the other presentation (19). Furthermore, the number of integrated foods will be reported as a treatment outcome based on parents’ report.

#### 2.9.2 Additional measures

Parents and Children will complete demographic characteristics assessment (e.g., gender, age, ethnicity) and psychiatric/medical history. Furthermore, we will assess comorbid diagnosis by both diagnostic interviews and the self-report measures assessing depression (Children’s Depression Screeners (46)) and anxiety levels (State–Trait Anxiety Inventory (STAI) (47)). Furthermore, we will record video therapy sessions to check for adherence of the study therapist to the intervention. See **Table 2** for a detailed overview including other variables of interest that are not part of the main analysis.

**Table 2:** Overview over measures and measuring points.

Instrument	Author (German-language version)	Construct	Number total items	Subscales	Scale	Caregiver					Children				
						Measuring points					Measuring points				
						0	1	2	3	4	0	1	2	3	4
Primary outcomes (Feasibility)															
Credibility/expectancy Questionnaire	(42)	Treatment expectancy and credibility	6	Expectancy; Credibility	9-point Likert scale from 1 = not at all to 9 = very; 11-point Likert scale from 0 to 100%		x	x	x						
Credibility/Expectancy	Adopted from (48)	Treatment expectancy	1	How sure are you that participating in ExpeditionFood will help you with your eating problems?	9-point Likert scale from 1 = not at all to 9 = very;							x	x	x	
Client satisfaction questionnaire, adapted for inpatient fields of work	(49)	Satisfaction with treatment service	8	/	4-point Likert scale; different scales (e.g., 1 = “excellent” to 4 “bad”			x	x				x	x	
Adverse Events	Adopted from (41)	Adverse events related to the intervention	3		1 Yes/no item; 1 open item to describe the event; 2 severity items on a 4-Point Likert scale 1= not at all to 4= very much		x	x	x	x		x	x	x	x
Secondary outcomes (Measures of ARFID)															
Eating Disorder Examination (EDE) ARFID module	(33)	DSM-5 based diagnosis of ARFID		Includes items on sensory sensitivity and psychosocial impairment	Expert-Rating: Criterion Present Yes/No, symptom severity 0-6		x		x			x		x	
Nine Item Avoidant/Restrictive Food Intake disorder screen (NIAS)	(35, 43) Translated with permission.	Symptom of ARFID	9	picky eating, appetite, fear	6-Point Likert scale 0 = Strongly Disagree to 5 = Strongly Agree.	x	x	x	x	x		x	x	x	x
Food Neophobia Scale (FNS) (shortened version)	(44, 45)	Food Neophobia	7		7-point Likert scale ranging from 1= “strongly disagree” to 7 =“strongly agree.”	x	x	x	x	x		x	x	x	x
Diagnostic Interview for Mental Disorders in Children and Adolescents	(36)	Diagnosis of comorbid symptoms (DSM-5)			Criterion fulfilled yes/partially/no		x								
Children’s Depression Screeners (ChilD-S)	(46)	Depression	8		4 Point Likert scale; 0 = “agree” to 3 = “disagree”							x	x	x	x
State–Trait Anxiety Inventory—Child version trait subscale	(47)	Trait Anxiety	20		3-response scale							x	x	x	x
Generic Quality of Life Instrument for Children-Revised-	(51)	Health-related quality of life	24	Physical well-being psychological wellbeing, self-esteem, family, friends, education/school	5-point Likert scale from 0 = “never” to 4 = “always”							x	x	x	x
Working alliance inventory—short revised	(52, 53)	Therapeutic alliance	12		5-point Likert scale from 1 = “rarely” to 5 “always”			x	x	x					
Therapeutic Alliance Scales for Children	(54)	Therapeutic alliance	12	Bond/working alliance; Positive and negative aspects of the emotional bond	4-Point Likert scale from 1 = “not at all” to 4 = exactly right”								x	x	x
Additional measures															
Children Eating Disorder Examination-Questionnaire (EDE-Q8)	(55, 56)	eating disorder psychopathology	8	restraint, eating concern, weight concern, and shape concern	0–6 scale = number of days in the previous 28; or (b) “Not at all” to “Markedly, ratio scale	x						x	x	x	x
Children Eating Disorder Examination-Questionnaire Parent version (EDE-QP-parent version)	(56)	eating disorder psychopathology	28	restraint, eating concern, weight concern, and shape concern	0–6 scale = number of days in the previous 28; or (b) “Not at all” to “Markedly, ratio scale	x									
The Affinity for Technology Interaction (ATI) Scale	(57)	Affinity for Technology Interaction	9		6-point Likert scale, 1 = “strongly disagree” to 6 = “strongly agree”		x								

ARFID, avoidant/restrictive food intake disorder. Assessment will take place at screening (t0), baseline (t1), mid-intervention (t2), post-intervention (t3) and 4-week follow-up (t4). Additionally, parents will be asked to fill out weekly the FNS; NIAS, and adverse event scale. Furthermore, number of integrated new foods will be tracked by caregivers from week 3 onwards. Demographic characteristics (e.g., gender, age) will be assessed at screening and baseline and parent-child interaction regarding food at pre-, mid and post-treatment.

### 2.10 Proposed statistical analysis

As our primary aim is to assess feasibility, we will report recruitment rates and retention rates and number of adverse events until post-assessment with descriptive statistics. We will also report feasibility ratings of parents regarding the duration of sessions and materials, number of foods tasted per week, as well as the overall therapy duration of individuals completing the intervention. Acceptability will be descriptively reported based on parents rating on the treatment credibility (Credibility/Expectancy Questionnaire (CEQ) (48) and treatment satisfaction (Client Satisfaction Questionnaire (CSQ) (49)), as well as single-items for children.

As a secondary aim we will analyze ARFID symptoms reduction with paired sample t-test for expert-rated psychosocial impairment and sensory sensitivity (based on the Eating Disorder Examination (EDE) ARFID module, as well as self-report measures of symptoms of ARFID (i.e., Nine Item Avoidant/Restrictive Food Intake disorder screen (NIAS) (43) and Food Neophobic Scale (FNS) (44)) and children’s quality of life will be conducted from pre- to post assessments and pre-to follow-up.

Exploratory, in line with previous research we will also analyze anxiety (STAI (47)) and depression levels (Children’s Depression Screeners (46)) using dependent t-tests to test for potential changes in comorbid symptoms (12, 15).

## 3 Discussion

The aim of this feasibility trial is to evaluate the feasibility and proof of concept of a therapist-guided cognitive therapy intervention for children with ARFID and their parents/guardians.

This ExpeditionFood intervention is based on the manualized CBT-AR treatment which has been shown to be feasible in a face-to face intervention format in children and adolescents (15). The intervention could help to overcome some of the treatment barriers (22, 23) for ARFID by making the CBT-AR more accessible in regions with less specialized treatment services and including mostly self-guided sessions allowing for more flexible timing of sessions.

The current design has several strengths. First of all, the digital format and emphasis on self-guided session allows families to be more flexible in their participation, increases accessibility and can reduce commute and family time constrains. Moreover, this is the first ARFID specific intervention translated to German making CBT-AR available in German speaking countries where ARFID specific interventions are lacking.

However, the current design also has some limitations. First, we opted to focus this standardized short intervention only on the sensory sensitivity presentation of ARFID. Although this will provide preliminary insight into the feasibility of a standardized digital intervention, it will also limit the generalizability of the findings to other presentations of ARFID. Additionally, we decided to focus on a rather young age range compared to previous CBT-AR trials, as parents/guardians might play a more central role than in older age ranges. Further extensions and adaptations may be needed for older adolescents and adults or for children with a neurodiversity, and might be tested in follow-up studies to this pilot trial. Further, multiple case reports have suggested that CBT-AR can be delivered with good outcomes to individuals with neurodivergent profiles (17, 50). To provide additional support for the personalized ARFID formulation and the administration of food exposures, we have incorporated video therapy sessions with a study therapist. While this prevents the stand-alone use of the digital intervention at the moment, the majority of the intervention can be still conducted in a self-guided manner. Similarly, we opted for non-standardized messages in the third intervention stage to help families addressing potential challenges related to food exposure. While this requires the availability of a study therapist and might reduce generalizability and may not be as economical as possible, it may also increase engagement in the intervention and reduce drop out. Lastly as this is the first intervention study in Germany and commonly used self-report measures (i.e., NIAS) assessing symptoms of ARFID are currently not yet validated in German, the findings will have to be interpreted bearing this limitation in mind.

## 4 Conclusion

ARFID is mostly common in children and is associated with severe health consequences. However, families with children with ARFID report various treatment barriers and long treatment journeys, suggesting a need for flexible and low-threshold interventions. Cognitive-behavioral therapy has shown to be feasible in face-to-face trials supporting proof-of-concept. However, intervention studies for ARFID and specialized treatment services in German-speaking countries are lacking. The present study will aim to investigate the feasibility of CBT-AR delivered as a therapist guided digital intervention and investigate the proof of concept in a parent-guided format. The present study has the potential to inform further clinical trials on digital delivery of interventions for ARFID.
